# Noise-tunable nonlinearity in a dispersively coupled diffusion-resonator system using superconducting circuits

**DOI:** 10.1038/srep41313

**Published:** 2017-01-25

**Authors:** Christin Rhén, Andreas Isacsson

**Affiliations:** 1 Chalmers University of Technology, Department of Physics, SE-412 96 Göteborg, Sweden

## Abstract

The harmonic oscillator is one of the most widely used model systems in physics: an indispensable theoretical tool in a variety of fields. It is well known that an otherwise linear oscillator can attain novel and nonlinear features through interaction with another dynamical system. We investigate such an interacting system: a superconducting LC-circuit dispersively coupled to a superconducting quantum interference device (SQUID). We find that the SQUID phase behaves as a classical two-level system, whose two states correspond to one linear and one nonlinear regime for the LC-resonator. As a result, the circuit’s response to forcing can become multistable. The strength of the nonlinearity is tuned by the level of noise in the system, and increases with decreasing noise. This tunable nonlinearity could potentially find application in the field of sensitive detection, whereas increased understanding of the classical harmonic oscillator is relevant for studies of the quantum-to-classical crossover of Jaynes-Cummings systems.

The harmonic oscillator is one of the most well-understood dynamical systems in physics, and is used as a model in nearly every field. The classical harmonic oscillator was studied already by Galileo Galilei, while its quantum counterpart was described in 1925 by Paul Dirac. It remains one of few models that can be exactly solved, and as such, it features prominently in courses on classical and quantum mechanics. It is perhaps surprising, then, that the harmonic oscillator still remains at the forefront of contemporary physics research.

Today, considerable attention is devoted to harmonic oscillators that interact with an auxiliary dynamical system. This situation appears, for instance, in circuit quantum electrodynamics^1^,^2^,^3^ and quantum information processing^4^,^5^,^6^,^7^,^8^, where the harmonic oscillator models a superconducting microwave circuit and the auxiliary system is a qubit. Then, manipulation of the circuit allows for control and read-out of the state of the qubit. In a similar manner, when the auxiliary system is a second harmonic oscillator, as in optomechanics^9^,^10^,^11^,^12^, one of the oscillators can be damped or driven by manipulating the other.

A case that exhibits a variety of intriguing, nonlinear effects is when another very common model system takes the role of auxiliary system: the diffusing Brownian particle. One proposed realization of such a coupled system is a diffusing particle loosely adsorbed on the surface of a nanomechanical resonator^13^,^14^,^15^,^16^,^17^. Then, the particle position directly influences the oscillator’s natural frequency, and the oscillator in turn provides an amplitude-dependent inertial back-action force on the particle. Despite its apparent simplicity, this diffusion-resonator system exhibits surprising effects such as induced nonlinearity^13^ and bistability^14^, inhomogeneous dephasing^15^, as well as mode coupling and non-linear dissipation^16^,^17^. As shown recently^18^, these features are rather generic for a harmonic oscillator mode coupled dispersively to an auxiliary dynamical system, under certain circumstances. However, with the current state of the art, it is very difficult to fabricate this nanomechanical system in a parameter regime where an interesting physical response will be observable.

Here we propose an alternative realization of a resonator-diffusion system, making use of superconducting circuit elements. These allow for a high degree of control over the relevant parameters, some of which can be tuned *in situ*. Our proposed realization, depicted in Fig. 1(a), makes use of a resistively shunted superconducting quantum interference device (SQUID), whose phase variable will act as a diffusing particle due to the presence of noise in the shunting resistor. The harmonic oscillator is represented by a lumped superconducting LC-resonator, and is inductively coupled to the SQUID. We find that when the resonator is driven, the SQUID phase takes one of two equilibrium values; it behaves as a classical two-level system. Interestingly, the LC-circuit exhibits dramatically different dynamics for the two values of the phase. In one case the circuit becomes a linear oscillator, while in the other it is highly nonlinear and can be multistable. As the resonator amplitude increases, the system switches between linear and non-linear regimes in a quasi-periodic manner, determined by the noise level and the drive amplitude. We derive an analytical model that is very successful at predicting the two regimes, and discuss where this model breaks down.

While it is clear that the tunable nonlinearity found in the studied circuit could find application in the field of sensitive detection (c.f. Josephson bifurcation amplifiers^19^), our results also have more fundamental implications. In the quantum regime, a two-level system coupled to a harmonic oscillator is described by the well-studied Jaynes-Cummings Hamiltonian. However, an understanding of the transition between this quantum regime and its classical counterpart remains elusive. As we here investigate the *classical* dynamics of a harmonic oscillator coupled to a two-level system, new light is shed on the less-known half of this quantum-to-classical crossover.

## Results

### Circuit description

**Figure 1:**
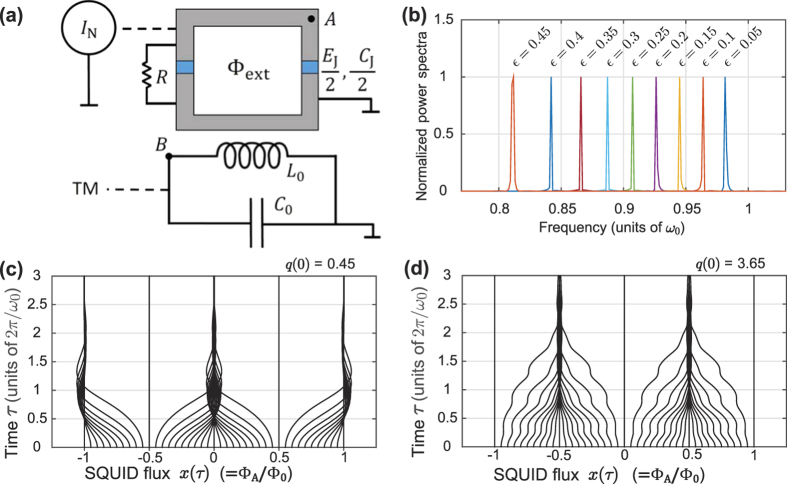
(**a**) Superconducting circuit realization of a diffusion-resonator system. A symmetric SQUID is inductively coupled to a lumped LC-resonator. A weak inductive coupling ensures a dispersive interaction between the two systems. Additional noise can be introduced in the system using an external noise current source *I*_N_. For read-out and actuation, the LC-resonator can be coupled inductively or capacitively to an external transmission line. The nodes *A* and *B* indicate where the node fluxes used below as dynamic variables are defined. (**b**)**–**(**d**) Numerical integration of the full equations of motion (5)–(6), absent external drive and noise ( *f* (*τ*) = 0, *γ* = 0, *η* = 0, and 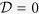). (**b**) Resonator power spectra for increasing coupling *ε*. The phase of the SQUID is rapidly trapped at integer **(c)** or half-integer (**d**) values of *x*, depending on resonator amplitude. Note that the quite large values of *ε* used in (**b**) are chosen to illustrate the frequency shift clearly; the *ε* = 0.01 used in (**c**)–(**d**) would correspond to a much smaller frequency shift.

We study a system consisting of a symmetric SQUID in the vicinity of a lumped LC-resonator with capacitance *C*_0_ and inductance *L*_0_, as shown in Fig. 1(a). For actuation and readout purposes, the resonator can be coupled to an external transmission line. Each Josephson junction in the SQUID is characterized by a capacitance *C*_J_/2 and Josephson energy *E*_J_/2. We take as dynamic variables the fluxes 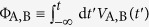 at points *A* and *B*, respectively, indicated in Fig. 1(a). The conservative dynamics of the system is then described by the Lagrangian


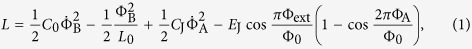


where Φ_0_ = *h*/2*e* is the flux quantum. The external flux Φ_ext_ threading the SQUID is partly determined by the current in the LC-circuit. If there are no other sources of external flux, the coupling is linear to lowest order in Φ_B_; Φ_ext_ ≈ 2*g*Φ_B_. Defining nondimensional variables *q* = 2*πg*Φ_B_/Φ_0_ and *x* = Φ_A_/Φ_0_, Eq. (1) leads to the equations of motion


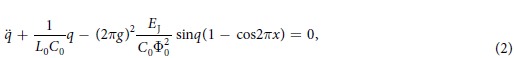



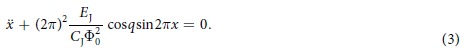


The SQUID has a normal total resistance *R*, arising from either junction-internal resistance or from additional shunting. That is, the equation for *x* should be supplemented by damping and noise, leading to the equation for the resistively and capacitively shunted Josephson junction (RCSJ) model^20^,^21^:


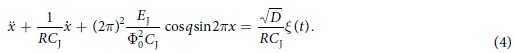


The RCSJ-model has reliably been able to reproduce experimental^22^ results in regimes where 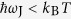, with 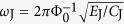 being the plasma frequency.

As noted already by Ambegaokar and Halperin^23^, the SQUID phase dynamics is that of a particle executing Brownian motion in a potential. For purely thermal fluctuations, the current noise in the resistive component is given by the Callen-Welton formula 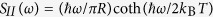. We consider here the classical limit 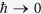, which allows us to treat the noise as Gaussian white noise: 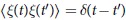 with a diffusion constant given by 
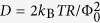
. However, it is also possible to impose noise externally by attaching a noisy current source *I*_N_, as shown in Fig. 1(a).

Rescaling the time variable to dimensionless time *τ* = *ω*_0_*t*, with 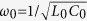, the equations of motion reduce to the form


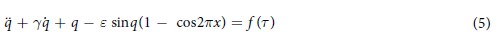



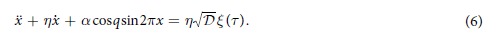


Here, we have introduced a finite quality (Q-) factor 1/*γ* to the LC-resonator, and added the external drive *f(τ*).

The nondimensional constants *ε, α, η*, and 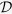 entering Eqs (5) and (6) are related to physical quantities through


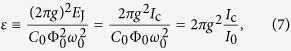



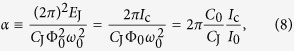



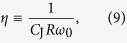



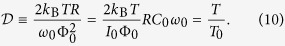


Here, 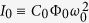 is the characteristic scale for the current through the LC-circuit, and *I*_c_ = 2*πE*_J_/Φ_0_ is the critical current of the junctions. The temperature scale is *T*_0_ = Φ_0_*I*_0_/2*k*_B_*RC*_0_*ω*_0_.

It should be noted that as the dissipative term 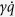 is introduced in Eq. (5), the fluctuation-dissipation theorem dictates that a stochastic force component 
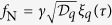
 is included in the external force *f*. This additive noise *f*_N_ is proportional to *γ* = 1/*Q*, which, in LC-circuits at cryogenic temperatures, can easily be as small as 10^−5^−10^−6^. Even for the comparably bad LC-resonator (with *γ* = 10^−3^) used in our simulations, the effect of the flux-noise term in Eq. (6) is an order of magnitude larger than that from *f*_N_ with the parameters used here.

#### Parameter values

We consider a typical LC-circuit with *L*_0_ = 1 nH and *C*_0_ = 0.1 pF, corresponding to an LC-frequency of 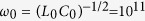 s^−1^. The resulting characteristic current scale for the LC-circuit is *I*_0_ = 2 μA. We further consider junctions with Josephson energy *E*_J_ = 0.6 meV and capacitance *C* = 1 pF, shunted by a resistance *R* = 16 Ω. Using an external shunt resistance, *I*_c_ becomes largely independent of *R*, and can be tuned by the temperature; here, we have *I*_c_ = 0.5 μA at a temperature *T* = 3 K. These parameter values, along with the resulting dimensionless parameters, are listed in Table 1.

For the nondimensional coupling constant *g* between the SQUID and the resonator we use *g* = 0.1, and set the LC-resonator inverse Q-factor to *γ* = 0.001. The relatively large values of *g* and *γ* were chosen for computational convenience. As will be seen in the analysis below, for weaker coupling *g* the same response will occur provided the resonator damping *γ* is reduced accordingly.

We will consider a periodic driving force 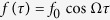, where *f*_0_ is dimensionless. In practice, this coefficient is related to the number of drive photons. The mean photon occupancy of the LC-resonator is


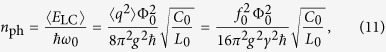


where the resonator energy 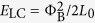 and 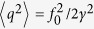 for an unperturbed resonator (*ε* = 0) driven at resonance. Substitution of the parameter values of Table 1 yields 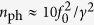. For an inverse Q-factor *γ* = 10^−3^ and drive amplitudes 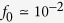, we thus find *n*_ph_ ≈ 10^3^.

#### Equivalence with nanomechanical system

To connect Eqs (5)–(6), to the nanomechanical resonator-physical particle system studied in refs 13, 14, 15, 16, 17, we note that for a small amplitude 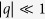, Equations (5) and (6) resemble the equations of motion of a particle diffusing on a vibrating string. Expanding the trigonometric terms and identifying the vibrational mode function 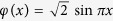, we find


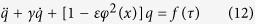



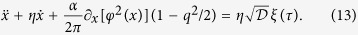


This is exactly the single-mode equations of motions seen in ref. 16, with the addition that the unperturbed (*q* = 0) motion of the particle described by *x* is no longer free diffusion. Instead, the unperturbed SQUID phase is affected by a spatial potential proportional to *φ*^2^(*x*). For *q* ≠ 0, *x* thus moves in an effective potential given by the combination of this spatial potential, and of the *q*-dependent “inertial” potential created by the resonator oscillation. With this addition taken into account, the nanomechanical system and the superconducting circuit show the same dynamics; the presence of a “particle” causes the frequency of the LC-resonator to shift downwards, as shown in Fig. 1(b), while the resonator motion causes trapping of the particle, see Fig. 1(c–d).

### Linear and nonlinear regimes

In the superconducting circuit, the total effective potential that determines the dynamics of the SQUID flux *x* is a combination of the effective potential created by the oscillation in the LC-circuit, that traps the flux near *x*_eq._ = *n* + 1/2, 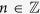, and a spatial potential proportional to sin^2^*πx*, that traps the flux near *x*_eq._ = *n*. The steady-state value *x*_eq._ thus depends on the relative strength of these two effects, which is determined by the resonator amplitude; see Fig. 1(c–d).

This interaction of two periodic potentials causes the resonator-SQUID system to display very interesting action-backaction dynamics. The resonator amplitude determines the equilibrium position *x*_eq._ of the flux particle. Both integer and half-integer *x*_eq._ have in common that the supercurrent through the SQUID, *I*_s_ = *I*_c_cos*q*sin2*πx*, vanishes, and Eq. (6) reverts to the familiar Langevin equation. However, the value of *x* has a dramatic impact on the dynamics of the resonator, tuning it from linear to highly nonlinear depending on whether *x*_eq._ is integer or half-integer.

When *x* is an integer, the term proportional to *ε* in Eq. (5) vanishes, and the equation for the LC-resonator becomes that of a driven damped linear oscillator. As such, it should exhibit a Lorentzian frequency response with maximum at *f*_0_/*γ* and width *γ*. When *x* is a half-integer, the absolute value of the *ε*-term is maximized; this is the maximally non-linear regime. For *f*_0_ = 0 and *x* = *n* + 1/2, Eq. (5) becomes 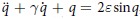, which describes an inverted physical pendulum of the kind used in the Holweck-Lejay gravimeter^24^.

### Driven response

We now turn to the driven response by considering a periodic driving force *f*(τ) = *f*_0_cosΩ*τ*. The resonator amplitude will depend on the drive amplitude and frequency, and the value of *x* will in turn depend on the resonator oscillation.

First, we analytically estimate the system’s response to the drive by analyzing it in the adiabatic, mean-field, rotating wave approximation. The full stochastic equations of motion are then numerically solved. Except for in a small region of anomalous response, the agreement between the analytical and numerical solutions is excellent.

#### Adiabatic RWA solution

To find the steady-state solution of the slow-moving envelope |*u*| of the resonator oscillation, we make the change of variables 2*q* = (*ue*^*i*Ω*τ*^ + *u*^∗^*e*^−*i*Ω*τ*^), 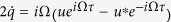. In the rotating wave approximation (RWA), the equations of motion (5)-(6) transform into


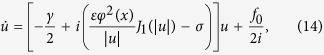



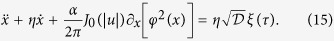


Here, we have assumed that the detuning *σ* = Ω − 1 is small (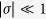), and we use 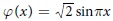 for brevity. Additionally, *J*_0,1_ are Bessel functions of the first kind. Note that the coupling constant *g* only affects *ε* in Eq. (14), and that a change in *ε* can be compensated for by a corresponding change in damping *γ*.

The dynamics captured by Eqs (14)–(15), 37 can also be described by the equivalent Fokker-Planck equation^25^ (FPE):


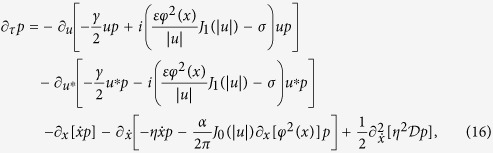


where 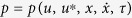 is the system probability density in state space. In the adiabatic limit, in which the relaxation time of the SQUID flux dynamics is much shorter than the relaxation time of the resonator (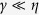), the system state can be approximately described by a quasi-stationary probability distribution 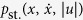. By treating the slow variable 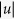 as a constant, we find from Eq. (16) that *p*_st._ is the solution of


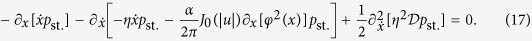


This can be interpreted as the equation of motion for a system described by the (non-dimensionalized) Hamiltonian 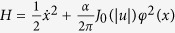, connected to a reservoir with dimensionless temperature 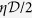. It follows that


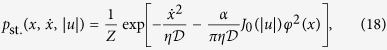


where 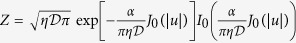, and *I*_0_ is the modified Bessel function of the first kind.

We now make the mean-field approximation 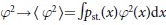, and solve for the stationary solution of Eq. (14). The result is that the stationary amplitude |*u*| is determined by


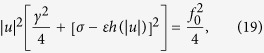


where the frequency shift *h*(|*u*|) is given by


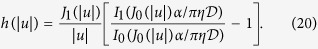


Interestingly, only the ratio *α/πη*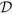 = *I*_c_Φ_0_/*k*_B_*T* = 2*I*_c_Φ_0_/*RS*_0_ (where *S*_*II*_(*ω*) = *S*_0_ for white noise) enters into the expression for the scaled frequency shift *h*(|*u*|). This ratio can also be written as 2*πE*_J_/*k*_B_*T*. In other words, the frequency shift, and hence the qualitative dynamics of the system, is determined by the ratio between the Josephson energy and the thermal energy.

**Figure 2:**
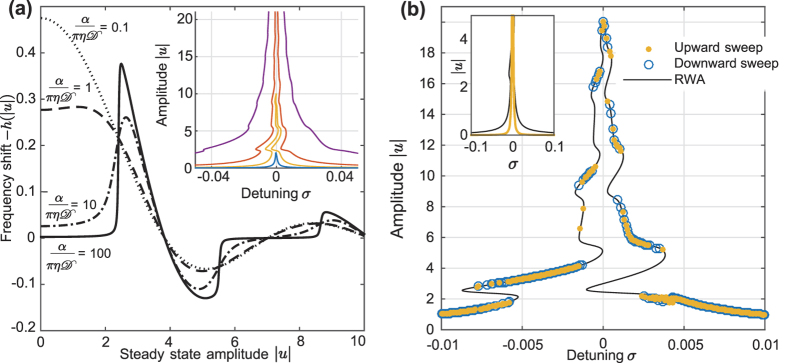
(**a**) Frequency shift *h*(|*u*|) as function of amplitude |*u*| in the adiabatic, mean-field, rotating wave approximation for different values of the ratio *α/πη*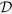. In the low noise limit (solid curve), for small |*u*| the phase particle is trapped at integer *x*, resulting in a zero frequency shift. For larger diffusion, the resonant frequency at low amplitudes increases with increasing noise (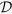). The inset shows the corresponding frequency response curves as a function of detuning *σ* = Ω − 1, obtained by solving Eq. (19). Here, *α/πη*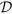 = 25, as given by the parameter values in Table 1, and we consider drive amplitudes *f*_0_ = 0.002 (blue curve), *f*_0_ = 0.01 (yellow curve), *f*_0_ = 0.04 (red curve), and *f*_0_ = 0.2 (purple curve). For moderate drive amplitudes 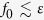, multistability beyond bistability is possible. (**b**) Simulated resonant response of the circuit, as the drive frequency is swept up (yellow dots) and down (blue circles). The black curve is the analytical response; the agreement is excellent. Here, the drive amplitude *f*_0_ = 0.02, and *α/πη*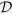 = 25. Since temperature is finite, there is noise-induced switching between multistable states, and hysteresis loops are smeared. The inset shows the bottom of the resonant peak (black) together with the Lorentzian response of an unperturbed system (yellow) – broadening is significant.

In Fig. 2(a), the frequency shift *h*(|*u*|) is shown for four different values of this ratio. For low resonator amplitudes (small |*u*|), the resonant frequency shifts downwards upon increasing the noise. As amplitude increases, either softening or hardening is observed depending on the noise power. The function *h* has an infinite number of crossings with the horizontal axis, tending to zero as |*u*|^−3/2^ in the limit of large |*u*|. The overall shape of the resonance curve, shown in the inset of Fig. 2(a), can be understood from treating the limits of high and low noise.

In the low noise limit, 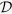 → 0, the “particle” coordinate *x* will localize at integer values (*x* = *n*) if *J*_0_(|*u*|) > 0, and at half-integer values (*x* = *n* + 1/2) if *J*_0_(|*u*|) < 0. The SQUID phase thus behaves as a classical two-level-system (TLS), whose state depends on the amplitude of the resonator. In this limit, the frequency shift *h*(|*u*|) approaches 2[*θ(J*_0_(|*u*|)) − 1]*J*_1_(|*u*|)/|*u*|. Hence, the zeros of *J*_0_ separate regions where the oscillator response is shifted away from the unperturbed Lorentzian line shape and regions where the two coincide: between regions where the LC-circuit has ordinary linear behavior and regions where it behaves as a driven Holweck-Lejay-like resonator.

As noise increases, one sees from Fig. 2(a) that the sharp features of the frequency shift *h*(|*u*|) are smoothed out, so that *h*(|*u*|) only vanishes at those discrete amplitudes |*u*| that correspond to zeros of *J*_1_. Hence, in the presence of non-negligible noise the piecewise linear behavior in |*u*| obtained for 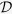 = 0 is destroyed, and for strong noise *h*(|*u*|) → −*J*_1_(|*u*|)/|*u*|. We conclude that by varying the noise intensity, the frequency response can be tuned.

The shape of the response curve is also influenced by the drive strength. As expected, and also shown in the inset of Fig. 2(a), the resonance peak looks Lorentzian for small *f*_0_, but quickly takes on a flame-like character as the driving force increases. However, as the drive amplitude increases further, the response once more resembles a Lorentzian. This return to apparent linearity can again be traced back to the structure of the frequency detuning function *h*(|*u*|), which decays algebraically with |*u*|. Consequently, the frequency shift near the top of the resonance peak quickly decays with increasing *f*_0_. At the base of the resonance, on the other hand, the width of the peak is of order *f*_0_/|*u*|, whereas the frequency detuning scales with *ε*. Hence, we expect no visible nonlinear response when 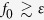.

### Frequency response

The stochastic equations of motion (5)–(6) were numerically integrated using a second-order algorithm^26^,^27^, with the parameter values listed in Table 1. These correspond to *α/πη*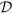 = 25.

To begin with, the resonant response of the LC-circuit was calculated. The drive frequency Ω was varied while *f*_0_ = 0.02, and the corresponding amplitude response found; the results are shown in Fig. 2(b). The agreement between simulation and the analytical results above is excellent.

**Figure 3:**
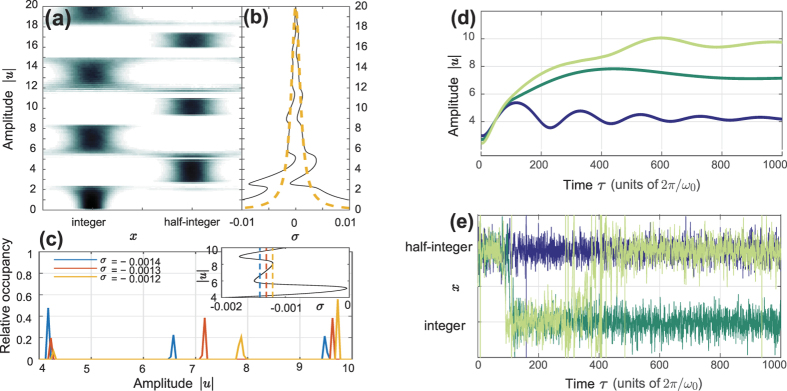
(**a**) Distribution of the SQUID phase *x* (mapped to the interval 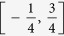) as a function of resonator amplitude |*u*|. **(b)** Theoretical response curve (solid black line) together with the unperturbed Lorentzian response (yellow dashed line). When *x* is an integer, the resonator decouples from the SQUID, and the resonator response is very close to the Lorentzian. For half-integer *x*, the magnitude of the coupling to the SQUID is maximized, and the resonator response is highly non-linear. **(c)** Distribution of resonator amplitudes for three values of the detuning *σ*. The inset shows a close-up of the relevant region of the analytical resonance curve, where the dashed lines indicate the examined values of *σ*. For a given detuning and drive power, three different amplitudes can be observed. **(d)** Three examples of the time evolution of |*u*| for *σ* = −0.0013. Despite quite small variations in the initial conditions, the system eventually settles in very different states. **(e)** The corresponding time evolutions of *x*. In each case, the SQUID phase is initially trapped at a half-integer value. The subsequent evolution of *x*_eq._ is dictated by the resonator amplitude |*u*|, in agreement with (**a**).

In order to further check the validity of the RWA-analysis above, we extracted the distribution of *x(τ*) for states stabilized at a certain envelope amplitude |*u*|. The result is shown in Fig. 3(a), where switching between integer and half-integer *x*_eq._ is clearly evident. The sections where no values are plotted are those |*u*| where no stable state could be found, due to that ∂|*u*|/∂*σ* → ∞. For comparison, Fig. 3(b) includes the theoretical response curve together with the Lorentzian *f*_0_*γ*^−1^(1 + *σ*^2^/*γ*^2^)^−1^. In agreement with the discussion above, there is a clear correspondence between integer *x*_eq._ and regions where the resonance curve is very close to the unperturbed Lorentzian, whereas half-integer *x*_eq._ coincide with highly nonlinear resonant response.

Due to the presence of thermal noise, in Fig. 2(b) expected hysteresis loops are smeared and there is very little difference between frequency sweeps up and down. Instead, the existence of multistability is proven by making a large number of measurements at the same detuning. To that end, several hundred trajectories were calculated, and the final resonator amplitude was recorded in each case. The initial state 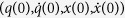 of the system was given by four random numbers, each uniformly distributed in the interval (−10,10). The result is shown in Fig. 3(c); the existence of multistability is clearly evident. Here, the considered values for the detuning *σ* were chosen to be such that the theoretical resonance curve indicates that several stable states might occur, as shown in the inset of Fig. 3(c).

The time evolution for the resonator amplitude and the SQUID phase is shown in, respectively, Fig. 3(d) and (e). Here, the detuning *σ* = −0.0013, and the initial state of the system is *x*(0) = 0.25, 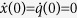, while *q*(0) is, respectively, 2.4, 2.6, and 3.0. As a consequence of the multistable response to driving, these small variations in initial amplitude can have a dramatic impact on the state in which the LC-circuit eventually settles. Indeed, Fig. 3(d) shows that all three states indicated in Fig. 3(c) do occur. In each case, the state of the *x*-TLS is governed by the amplitude |*u*|, as shown in Fig. 3(e). The phase is initialized equidistantly from the two *x*_eq._, but is trapped near *x*_eq._ = 1/2 within a few resonator cycles. Once trapped, the dynamics of the phase remains that of Brownian motion in a potential well, but the position of the well is a function of resonator amplitude. This is particularly clear in the case where *q*(0) = 2.4 (light green line); the resonator amplitude begins in a nonlinear regime, and monotonically increases through a linear region and back to non-linear. At the same time, *x* switches from being trapped at a half-integer value, to integer trapping, and back to non-integer. Note, however, that while the |*u*|-evolution is smooth and quite slow, the corresponding shifts between the two *x*_eq._ are very rapid, illustrating the TLS-character of the SQUID phase *x*.

### Zero-temperature limit

**Figure 4:**
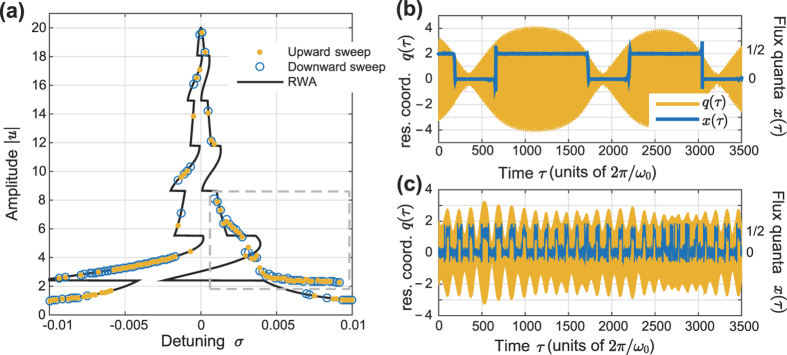
(**a**) Resonant response at *T* → 0 K. The transitions between linear and nonlinear regimes are much sharper than in the case of finite temperatures. The dashed box indicates the anomalous region of detuning *σ*, where analytical and numerical results do not coincide. **(b)–(c)** Time evolution for the LC-resonator amplitude *q* and the SQUID flux *x*, at *σ* = 0.003 and (**b**) *T* → 0 K (**c**) *T* = 3 K. The response in the anomalous region is non-stationary, with resonator amplitude beats caused by *x* switching between integer and half-integer values.

Finally, we consider the limit of millikelvin temperatures, such that 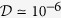. With all other parameters as in Table 1, then 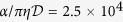. Consequently, the frequency shift *h*(|*u*|) exhibits incredibly sharp features for |*u*| such that *J*_0_(|*u*|) ≈ 0. The theoretical resonance curve inherits these sharp features, as can be seen in Fig. 4(a). Still, for a large part of the response curve, the calculated response fits the theoretical curve surprisingly well. The exception is an anomalous region of positive *σ*, indicated in Fig. 4(a) by a dashed box.

A typical time evolution of the oscillator coordinate *q* and the flux particle position *x* for detuning *σ* = 0.003 is shown in Fig. 4(b). As can be seen, here in the anomalous part of the response, the system makes quasiperiodic transitions between integer and half-integer values of *x*, leading to beats in the resonator amplitude. The beats stem from the appearance of transient frequency components at *εh*(|*u*|). These transients appear whenever a transition from integer to half-integer *x* occurs, that causes the resonance to abruptly shift downwards. While the system remains at half-integer *x* it is strongly nonlinear and can mix frequency components. Mixing with the drive at Ω ≈ 1 − *εh*(|*u*|) then causes a resultant which is on resonance, that consequently drives the oscillator at a shifted frequency, leading to the amplitude beats.

Note that the corresponding phenomena cannot occur for negative detuning *σ* < 0. Although the opposite process (half-integer to integer *x*) will lead to transients with positive frequency components, integer *x* puts the resonator in the completely linear regime. Frequency mixing is then absent, and no component resurrecting the off-resonant motion can appear. Instead, only transient switching behavior is seen before the system reaches a stationary oscillatory state 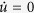.

This anomalous region of deviation between analytical and numerical results is seen also in Fig. 2(b), but only as less well-fitting data points near *σ* = 0.003. In this case, the smoothing of *h*(|*u*|) that is caused by the higher temperature makes the dynamics far less dramatic. While driving near *σ* = 0.003 will still cause *q(τ*) to contain frequency components with negative detuning, thermal noise will smear the resulting amplitude beats, as seen in Fig. 4(c). The time the system spends in the nonlinear regime is thus greatly decreased, and the frequency mixing is limited correspondingly. The higher noise level thus acts to *stabilize* the resonator dynamics. This hints at the presence of stochastic resonance, in the broad sense of “randomness that makes a nonlinearity less detrimental to a signal”^28^.

## Discussion

With superconducting circuit quantum electrodynamics being routinely done in the lab, the proposed system should be readily realized. Although the multistable response will only be visible for a particular range of drive powers, and the nonlinear parts of the resonance peak are very narrow, the current state of the art has matured to the point where detecting both these features is well within reach. A successful verification of the results in this Article would be the first experimental observation of induced nonlinearity in a diffusion-resonator system. Such an observation could stimulate further research into the influence of classical and quantum fluctuations in the interplay between harmonic oscillators and other dynamical systems.

## Additional Information

**How to cite this article**: Rhén, C. and Isacsson, A. Noise-tunable nonlinearity in a dispersively coupled diffusion-resonator system using superconducting circuits. *Sci. Rep.* **7**, 41313; doi: 10.1038/srep41313 (2017).

**Publisher's note:** Springer Nature remains neutral with regard to jurisdictional claims in published maps and institutional affiliations.

## Figures and Tables

**Table 1:** Typical values for circuit parameters, and the corresponding dimensionless parameters.

Physical parameters				Dimensionless quantities	
*T*_0_	1000 K	*E*_J_	0.6 meV	*γ*	0.001
*L*_0_	1 nH	*R*	16 Ω	*ε*	0.015
*C*_0_	0.1 pF	*C*_J_	1 pF	*α*	0.15
*ω*_0_	100 GHz	*ω*_J_	3 GHz	*η*	0.625
*I*_0_	2 μA	*I*_c_	0.5 μA	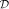	0.003

The simulation temperature *T* was chosen as 3 K, and the Q-factor of the LC-resonator was set to 1000.
